# Production of biopolymer precursors beta-alanine and L-lactic acid from CO_2_ with metabolically versatile *Rhodococcus opacus* DSM 43205

**DOI:** 10.3389/fbioe.2022.989481

**Published:** 2022-10-07

**Authors:** Laura Salusjärvi, Leo Ojala, Gopal Peddinti, Michael Lienemann, Paula Jouhten, Juha-Pekka Pitkänen, Mervi Toivari

**Affiliations:** ^1^ VTT Technical Research Centre of Finland Ltd., Espoo, Finland; ^2^ Department of Bioproducts and Biosystems, Aalto University, Espoo, Finland; ^3^ Solar Foods Ltd., Espoo, Finland

**Keywords:** carbon dioxide, lithoautotrophic, hydrogen-oxidizing bacteria, Rhodococcus opacus, L-lactic acid, beta-alanine, gas fermentation

## Abstract

Hydrogen oxidizing autotrophic bacteria are promising hosts for conversion of CO_2_ into chemicals. In this work, we engineered the metabolically versatile lithoautotrophic bacterium *R. opacus* strain DSM 43205 for synthesis of polymer precursors. Aspartate decarboxylase (panD) or lactate dehydrogenase (ldh) were expressed for beta-alanine or L-lactic acid production, respectively. The heterotrophic cultivations on glucose produced 25 mg L^−1^ beta-alanine and 742 mg L^−1^ L-lactic acid, while autotrophic cultivations with CO_2_, H_2_, and O_2_ resulted in the production of 1.8 mg L^−1^ beta-alanine and 146 mg L^−1^ L-lactic acid. Beta-alanine was also produced at 345 μg L^−1^ from CO_2_ in electrobioreactors, where H_2_ and O_2_ were provided by water electrolysis. This work demonstrates that *R. opacus* DSM 43205 can be engineered to produce chemicals from CO_2_ and provides a base for its further metabolic engineering.

## 1 Introduction

Climate change and global warming necessitate a shift to carbon-neutral chemical manufacture. Consequently, interest in development of bioprocesses employing autotrophic microbial hosts for production of chemicals from CO_2_ is constantly growing (Liu et al., 2020). By virtue of metabolic engineering, autotrophic microorganisms can be equipped with synthetic production routes for conversion of CO_2_ into various valuable compounds such as material precursors, flavors and biofuels (Hu et al., 2019). Aerobic and autotrophic species of hydrogen-oxidizing bacteria are particularly attractive production hosts because, in contrast to, e.g., photosynthetic organisms, they are able to assimilate CO_2_ under dark conditions. These bacteria, also called Knallgas bacteria, employ either the Calvin-Benson-Bassham (CBB) cycle or the reductive tricarboxylic acid cycle for CO_2_ fixation and they gain energy from H_2_ oxidation by the action of hydrogenases (Arai et al., 2010; Emerson and Stephanopoulos, 2019). The substrate H_2_ can be generated, e.g., by water electrolysis using renewable energy (solar or wind) (Nikolaidis and Poullikkas, 2017). Aerobic hydrogen-oxidizing bacteria couple the electrons from oxidation of H_2_ to the electron transfer chain with O_2_ as the final electron acceptor for the respirative adenosine triphosphate (ATP) generation. Therefore, these bacteria can generate more energy to produce biomass and complex natural products such as polyhydroxyalkanoates compared to, e.g., acetogenic bacteria with the anaerobic Wood-Ljungdahl pathway for CO_2_ fixation (Teixeira et al., 2018; Brigham, 2019).

The Gram-negative hydrogen-oxidizing bacterium *Cupriavidus necator* H16 is the best-characterized species of aerobic facultative lithoautotrophs. It can be considered an emerging autotrophic production platform and has been explored by several metabolic engineering studies (Yu, 2018; Brigham, 2019) for the production of value-added compounds such as isopropanol (Grousseau et al., 2014), 2-hydroxyisobutyric acid (Przybylski et al., 2015), methyl ketones (Müller et al., 2013), alkanes (Crépin et al., 2016), *α*-Humulene (Krieg et al., 2018), isobutanol (Chakravarty and Brigham, 2018) and acetoin (Windhorst and Gescher, 2019). Moreover, the recent studies demonstrated that the energy efficiency of CO_2_ fixation *via* the CBB cycle can be improved and the product titers increased by different metabolic engineering approaches and by optimizing the fermentation process and bioreactor design (Bommareddy et al., 2020; Garrigues et al., 2020; Li et al., 2020).

However, although several species of hydrogen-oxidizing bacteria have been identified, much of their metabolic potential remain untapped because the research and production strain development have concentrated on the species *C. necator* H16 (Brigham, 2019). The facultative lithoautotrophic strain *R. opacus* DSM 43205 (formerly referred to as *Nocardia opaca* 1b) is metabolically different from *C. necator* H16 and thus an interesting alternative host for conversion of CO_2_ to chemicals (Probst and Schlegel, 1973; Aggag and Schlegel, 1974; Klatte et al., 1994)*.* Unlike *C. necator* H16, that uses polyhydroxybutyrate as a carbon sink, *R. opacus* naturally accumulates fats (i.e., triacylglycerols), is Gram-positive, and contains only a cytoplasmic NAD^+^-reducing hydrogenase but no membrane-bound isoenzyme (Aggag and Schlegel, 1974; Schneider and Schlegel, 1977). The cytoplasmic hydrogenase is, however, very similar to the one of *C. necator* H16 with respect to its catalytic and molecular properties (Schneider et al., 1984). Both enzymes consist of four major subunits harboring a bound catalytic [NiFe] center along with several iron-sulfur clusters and monomeric flavin mononucleotides bound in the auxiliary subunits. The enzymes required for lithoautotrophic growth of *R. opacus* DSM 43205 include the cytoplasmic hydrogenase and ribulose-1,5-bisphosphate carboxylase (RuBisCo) that are encoded on the linear extrachromosomal conjugative plasmid pHG201 (Kalkus et al., 1990; Grzeszik et al., 1997). This genetic feature is shared between *R. opacus* DSM 43205 and *C. necator* H16 of which the latter contains these lithoautotrophy-related genes on the megaplasmid pHG1 (Schwartz et al., 2003).

**FIGURE 1:**
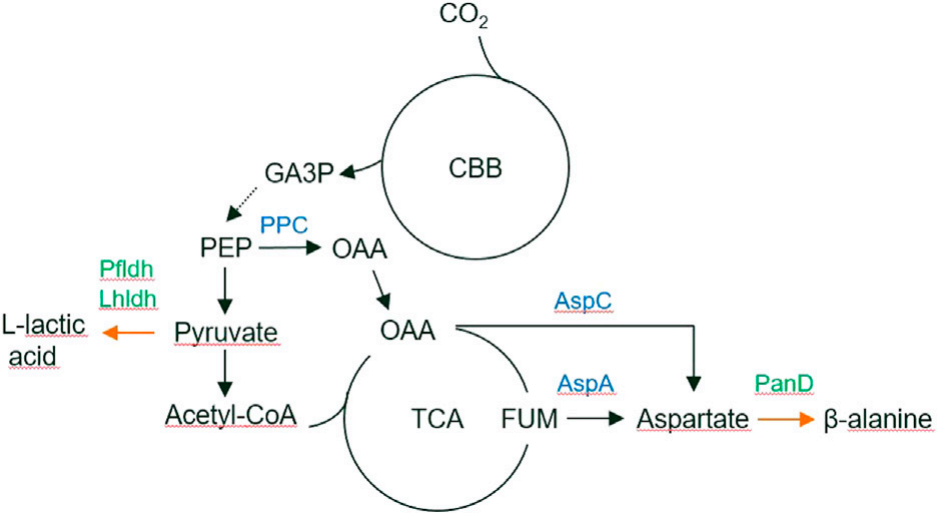
The biosynthetic pathways for L-lactic acid and beta-alanine production from CO_2_. Overexpressed genes are marked by green. Abbreviations: CBB, Calvin–Benson–Bassham cycle; GA3P, glyceraldehyde 3-phosphate; PEP, phophoenolpyruvate; OAA, oxaloacetate; TCA, tricarboxylic acid cycle; FUM, fumarate; PPC, phosphoenolpyruvate carboxykinase; Pfldh, *P. falciparum* L-lactate dehydrogenase; Lhldh, *L. helveticus* L-lactate dehydrogenase; PanD, *C. glutamicum* aspartate 1-decarboxylase.

Heterotrophic *R. opacus* strains have been studied due to their oleaginous metabolism and versatile biodegradation pathways (Holder et al., 2011; Henson et al., 2018). Genetic engineering tools are established for *R. opacus* (DeLorenzo et al., 2018) and used, e.g., for the production of fatty acids, wax esters and alkanes (Huang et al., 2016; Lanfranconi and Alvarez, 2017; Kim et al., 2019). In the present work, we engineered the autotrophic *R. opacus* DSM 43205 strain to convert CO_2_ into beta-alanine and L-lactic acid by expressing heterologous genes encoding aspartate decarboxylase and L-lactate dehydrogenase, respectively. Beta-alanine is a desired precursor for the synthesis of different polymers in the chemical industry (i.e., polyacrylamide, polyacrylate, polyacrylonitrile, and nylon-3) (Könst et al., 2009; Steunenberg et al., 2013; Ko et al., 2020). In addition, beta-alanine has commercial relevance as a nutritional supplement and is a precursor of pantothenate (vitamin B_5_), coenzyme A and pharmaceutical drugs (Khan et al., 2010; Trexler et al., 2015). In prokaryotes, beta-alanine is mainly produced by decarboxylation of L-aspartate by L-aspartate 1-decarboxylase (Cronan, 1980) (Figure 1). Extensive metabolic engineering of *Escherichia coli* and *Corynebacterium glutamicum* has already resulted in strains producing 85 g/L and 56 g/L beta-alanine from glucose, respectively (Wang et al., 2021; Li et al., 2022).

L-lactic acid, selected as a second production target, is used as a precursor for the production of polymers and has multiple applications in chemical and other industries (Eiteman and Ramalingam, 2015). Several companies have already successfully commercialized its heterotrophic microbial production that involves a single-step enzymatic reduction of pyruvate by lactate dehydrogenase (Figure 1). Bacterial and yeast hosts and their engineering for L-lactic acid production has been recently reviewed (Abedi and Hashemi, 2020). In addition, several other products can be envisioned for storing CO_2_ in the future, e.g., accumulation intracellular lipids (Kim et al., 2019), by the autotrophic *R. opacus* DSM 43205.

In this study, the autotrophic production of beta-alanine and L-lactic acid from CO_2_ and H_2_ as sole carbon and energy sources, respectively, is demonstrated using engineered derivatives of the *R. opacus* strain DSM 43205. Heterologous genes encoding aspartate decarboxylase (for beta-alanine production) or lactate dehydrogenase (for lactic acid production) were expressed from plasmid and the autotrophic production of both polymer precursors was performed by gas fermentation during which H_2_ was fed into the cultivation alongside with air and CO_2_ (Yu, 2018). Beta-alanine was additionally synthesized in a bioreactor where H_2_ was produced electrolytically *in situ* at a submerged cathode with concomitant oxygen production at an anode (referred here as electrobioreactor) (Schlegel and Lafferty, 1965). Moreover, in order to facilitate further development of this autotrophic production host, *R. opacus* DSM 43205 was sequenced by employing a combination of the long and short read technologies, leading to an improved genome assembly with higher coverage and fewer contigs as compared to the previously published genome (Sangal et al., 2016). The assembled genome was further used to construct a genome-scale stoichiometric metabolic model for *R. opacus* DSM 43205. The model was utilized to simulate the carbon and energy requirement for beta-alanine and lactic acid production with respect to growth.

## 2 Materials and methods

### 2.1 Genome sequencing, assembly, and annotation

Sequencing libraries were prepared by BaseClear BV (Leiden, Netherlands), using an Illumina Nextera XT kit for short-read sequencing and a 10 kb PacBio library preparation technique for long-read sequencing. BaseClear BV (Leiden, Netherlands) performed short-read Illumina-based paired-end sequencing (HiSeq 2500, 2x125bp) at a depth of 200 Mb, and long-read sequencing using the PacBio Sequel SMRT platform. BaseClear BV (Leiden, Netherlands) performed the quality filtering and delivered the resulting filtered raw data of the sequence reads. We used FastQC (version 0.11.7) for analysing the quality of raw sequencing reads to confirm that the short-read data showed high base-call quality across all the bases. As the PacBio Sequel platform does not report the quality values for the base calls, the long-read data was excluded from the *post-hoc* FastQC-based quality analysis. *De novo* genome assembly was performed using Unicycler v0.4.6 (Wick et al., 2017), utilizing the combination of the PacBio-derived long reads and Illumina-derived short reads. Gene prediction was performed using the bacterial genome annotation pipeline Prokka v1.14.5 (Seemann, 2014), and functional annotations were performed using eggNOG (Huerta-Cepas et al., 2016) and Pannzer (Törönen et al., 2018). The metabolic functional annotations were visualized together with the metabolic annotations of *R. opacus* strain PD630 (obtained from KEGG) using iPATH3 (https://pathways.embl.de/). The annotated genome sequence was submitted to European Nucleotide Archive (ENA) (Harrison et al., 2021) in the project with the accession number of PRJEB45460.

### 2.2 Genome-scale metabolic model reconstruction and simulations

A genome-scale metabolic model for *R. opacus* DSM 43205 was reconstructed using the automated reconstruction tool CarveMe (Machado et al., 2018). The bacterial universal metabolic model constructed based on the reactions obtained from BiGG models database (King et al., 2016) was used as the reference. The proteome sequence of *R. opacus* DSM 43205 derived in the gene prediction step was used to calculate the reaction scores for the model reconstruction as follows. The *R. opacus* DSM 43205 protein sequences were aligned to the sequences of the BiGG genes using Diamond (Buchfink et al., 2014) and the best alignment score for each BiGG gene was used as gene-level score. The gene-level scores were converted to reaction-level scores *via* the gene-protein-reaction rules as described in detail by (Machado et al., 2018). Briefly, the protein-level scores were calculated as the average gene-level score of all subunits in each protein complex, and the maximum of the protein-level scores of all isozymes that catalyze each reaction was used as the reaction-level score. The reaction-level scores were normalized with the median reaction score. Enzyme-catalyzed reactions without genetic evidence were given a score of −1 and the spontaneous reactions were assigned a score of zero. The *R. opacus* DSM 43205 genome-scale metabolic model reconstruction was performed with the python-based metabolic modelling package framed (https://github.com/cdanielmachado/framed), using the IBM ILOG CPLEX LP-solver v. 12.8.0 function *cplexlp*. The model simulations were performed using *cplexlp* and package *cobra* v. 0.20.0, and processed using package *numpy* v. 1.19.5. Flux distributions in optimal H_2_ limited growth, beta-alanine, and lactic acid production were simulated using parsimonious flux balance analysis and visualized on the KEGG map of Microbial metabolism using iPATH3 (https://pathways.embl.de/). Relative flux values were reflected by the arrow thickness with the exception that all fluxes less than or equal to 0.04 relative to H_2_ utilization were represented as 0.04 relative to H_2_ utilization for the visualization.

### 2.3 Strain and plasmid construction

Synthetic genes encoding aspartate 1-decarboxylase (*panD*) of *C. glutamicum* (NF003947.0) and lactate dehydrogenases of *Plasmodium falciparum* (Pf*ldh*) (WP_074506212.1) and *Lactobacillus helveticus* (Lh*ldh*) (WP_012211363.1) were ordered from Thermo Fisher Scientific. All three genes were optimized for expression in *R. opacus* and the optimized gene sequences are listed in Supplementary Material S1, Table 1. The plasmids pDD57 and pDD65 were kindly provided by Drew M. DeLorenzo (Washington University in St. Louis, MO) (DeLorenzo et al., 2018). pDD65 is an empty plasmid with a kanamycin marker and pAL5000 (S) backbone (Ranes et al., 1990) that was used to construct pDD57 by insertion of the gene *gfp*+ encoding a modified green fluorescent protein under the control of a strong constitutive promoter of *Streptomyces lividans* TK24 (DeLorenzo et al., 2018). In this study, *gfp*+ in pDD57 was replaced with either *panD*, Pf*ldh* or Lh*ldh* by releasing *gfp*+ with *Nde*I + *BamH*I digestion and using the remaining plasmid backbone in Gibson assembly cloning with *panD*, Pf*ldh*, or Lh*ldh* (Gibson et al., 2009). The resulting expression vectors for *panD*, Pf*ldh*, and Lh*ldh* and the empty vector pDD65 were introduced into *R. opacus* cells by electroporation. This was done by first growing *R. opacus* in 50 ml of tryptic soy broth overnight at 30°C followed by a cell harvest by centrifugation. The cells were washed twice with cold 20 mM HEPES buffer (pH 7.2) containing 15% glycerol and once with 5 mM HEPES (pH 7.2) containing 15% glycerol. Then, cells were suspended in 800 μl of 5 mM HEPES buffer (pH 7.2) containing 15% glycerol after which 1 μg of plasmid DNA was added to 80 μl of the cell suspension, mixed and subjected to electroporation in 1-mm cuvettes with the following settings: 25 μF, 400 Ω, 2.5 kV. Subsequently, the cells were incubated at 30°C for 3 h in 800 μl of super optimal broth with catabolite repression (SOC medium) before plating them on tryptic soy agar (TSA) plates containing 50 μg ml^−1^ kanamycin. The presence of expression vectors in the resulting *R. opacus* transformant strains ROP-pDD65 (ctrl), ROP-PfLdh, ROP-LhLdh, and ROP-PanD was verified by colony PCR using the plasmid-specific DNA oligomers and DreamTaq DNA polymerase (Thermo Fisher Scientific). The constructed plasmids and strains are listed in Table 1.

**TABLE 1:** Plasmids and strains used in this study.

Plasmids	Description	Reference
pDD65	*R. opacus* empty expression containing the pAL5000 (S) vector backbone and a kanamycin marker	Ranes et al. (1990)
pDD57	Derivative of pDD65 containing *gfp*+ under a strong constitutive promoter of *S. lividans* TK24	DeLorenzo et al. (2018)
pDD57-*panD*	Derivative of pDD57 with *gfp+* substituted with *panD* encoding aspartate decarboxylase of *C. glutamicum* (NF003947.0)	This study
pDD57-Pf*ldh*	Derivative of pDD57 with *gfp+* substituted with *ldh* encoding lactate dehydrogenase of *P. falciparum* (WP_074506212.1)	This study
pDD57-Lh*ldh*	Derivative of pDD57 with *gfp+* substituted with *ldh* encoding lactate dehydrogenase of *L. helveticus* (WP_012211363.1)	This study
**Strains**		
ROP-pDD65	*R. opacus* DSM 43205 harboring empty pDD65 (control strain)	This study
ROP-PanD	*R. opacus* DSM 43205 harboring pDD57-*panD*	This study
ROP-PfLdh	*R. opacus* DSM 43205 harboring pDD57-Pf*ldh*	This study
ROP-LhLdh	*R. opacus* DSM 43205 harboring pDD57-Lh*ldh*	This study

### 2.4 Growth media

All cultivations were carried out on a modified DSM-81 mineral medium (www.dsmz.de/microorganisms/medium/pdf/DSMZ_Medium81.pdf). The major chloride salts in the original recipe were replaced with the corresponding sulfates to reduce the formation of chlorine gas during electrolysis in electrobioreactor cultivations. Additionally, the vitamins were omitted from the recipe since they did not enhance bacterial growth (data not shown). Furthermore, beta-alanine is a precursor of one of the B vitamins (pantothenate) and therefore the presence of pantothenate in medium could interfere with the beta-alanine synthesis. The final medium composition per 1 L was: 2.3 g KH_2_PO_4_, 2.9 g Na_2_HPO_4_·2H_2_O, 5.45 g Na_2_SO_4_, 1.19 g (NH_4_)_2_SO_4_, 0.5 g MgSO_4_·7H_2_O, 11.7 mg CaSO_4_·2H_2_O, 4.4 mg MnSO_4_·H_2_O, 5 mg NaVO_3_, 0.5 g NaHCO_3_, 5 mg ferric ammonium citrate, 0.5 mg ZnSO_4_·7H_2_O, 1.5 mg H_3_BO_3_, 1 mg CoCl_2_·6H_2_O, 50 μg CuCl_2_·2H_2_O, 0.1 mg NiCl_2_·6H_2_O, 0.15 mg Na_2_MoO_4_·2H_2_O. 50 μg ml^−1^ kanamycin and/or 20 g L^−1^ glucose were added when appropriate.

### 2.5 Inocula and shake flask cultivations

*R. opacus* transformants were maintained on TSA plates containing 50 μg ml^−1^ kanamycin and in each case three individual transformants were studied. Inocula for glucose cultivations were grown at 30°C in 10 ml of modified DSM-81 media supplemented with 20 g L^−1^ glucose and 50 μg ml^−1^ kanamycin in 50 ml Erlenmeyer flasks. Shake flask cultivations performed with glucose as carbon source were carried out in 250 ml Erlenmeyer flasks in 50 ml of the same medium that was used to grow the inocula. Cultivations were started from optical density OD_600nm_ of 0.1 and incubated at 30°C with 220 rpm shaking. Inocula for shake flask cultivations and for cultivations in electrobioreactors both supplemented with CO_2_ as carbon source were prepared by transferring a loop-full of cells from a TSA plate into 100 ml Erlenmeyer flasks containing 20 ml of modified DSM-81 media supplemented with 50 μg ml^−1^ kanamycin. Precultures were incubated in a sealed container with 130 rpm shaking into which a gas mix (49% N_2_, 25% CO_2_, 13% H_2_, and 13% O_2_) was fed at a flow rate of 32 ml min^−1^ until OD_600nm_ was 4–5. Shake flask cultivations were started from OD_600nm_ of 0.1 and carried out in 20 ml of same media and under the same conditions as precultures.

### 2.6 Electrobioreactor cultivations

Electrobioreactor cultivations were performed in MR-1194 Bulk Electrolysis cell vials (100 ml; BASi, West Lafayette, IN) with custom-made Teflon lids as previously described by Nyyssölä et al. (2021). *R. opacus* preculture was added to 70 ml of modified DSM-81 medium supplemented with 13 μl of Componenta VO antifoam (Ecolab, Oegstgeest, Netherlands) to an OD_600nm_ of 0.2. A filter-sterilized gas mix consisting of 20% CO_2_ and 80% N_2_ (AGA, Espoo, Finland) was humidified by bubbling through sterilized water before sparging it into the reactor liquid at a flow rate of 6 ml min^−1^. The reactor temperature was maintained at 30°C with water circulated through the heating jacket of the reactor using two external water baths (Julabo, Seelbach, Germany and VWR International, Radnor, PA, United States). The reactor liquid was agitated by magnetic stirring at 400 rpm. A coiled titanium wire coated with a thin layer of iridium oxide (Ø 1.5 mm, Magneto Special Anodes, Schiedam, Netherlands) was used as anode and a coiled stainless steel capillary (Ø 1.6 mm, 316L-SS, Pfeiffer Vacuum GmbH, Asslar, Germany) was used as cathode with surface areas of 12.3 and 14.1 cm^2^, respectively. The voltage and current were controlled using a Wavenow potentiostat (Pine Research Instrumentation, Grove City, PA), and the AfterMath software (Version 1.3.7060, Pine Research Instrumentation). *In situ* water electrolysis was performed at a current of 18 mA (chronopotentiometry).

### 2.7 Analytical methods

#### 2.7.1 Light absorbance measurements to monitor cell growth

Biomass growth was measured from cultivations by taking 1–2 ml samples and measuring its optical density at *λ* = 600 nm (OD_600nm_) using a UV-1201, UV-vis spectrophotometer (Shimadzu, Kyoto, Japan). Highly dense samples were diluted to obtain an OD_600nm_ in the range of 0.1–0.3. The cell dry weight (CDW) was measured from 2 ml cultivation samples by separating the cells from the soluble culture fraction by centrifugation and washing them twice with MilliQ water before drying at them 105°C overnight. Alternatively, the CDW per litre of culture broth was calculated from the OD_600nm_ of the culture using a previously determined standard curve.

#### 2.7.2 Quantification of glucose and L-lactic acid by high-performance liquid chromatography

The extracellular concentrations of glucose and L-lactic acid from glucose-supplemented shake flask cultivations were determined by high-performance liquid chromatography (HPLC) on Fast acid and Aminex HPX-87H columns (BioRad Laboratories, Hercules, CA) with 2.5 mM H_2_SO_4_ as eluant and a flow rate of 0.5 ml min^−1^. The column was maintained at 55°C and analyte elution was detected using a Waters 410 differential refractometer and a Waters 2487 dual wavelength UV (210 nm) detector (Waters, Milford, MA).

#### 2.7.3 Quantification of L-lactic acid by combined gas chromatography and mass spectrometry

Extracellular concentration of L-lactic acid from CO_2_-cultivations was analysed using an 6,890 gas chromatograph combined with a 5,973 mass selective detector (Agilent, Santa Clara, CA). Each cell culture supernatant sample (50 μl) was spiked with internal standard (10 μl of 3-hydroxybutyric acid-1,3-13C2 acid) and the sample was evaporated into dryness under N_2_ flow. The dried residues were derivatized with a mixture of 50 µl of pyridine and 50 µl of N-Methyl-N-(trimethylsilyl) trifluoroacetamide reagent containing 1% of trimethylchlorosilane as a catalyst (70°C, 60 min). The injector (injection volume 1 µl) and inlet temperature was 250°C, and the oven temperature was increased from 50°C to 310°C. The analyses were performed on an DB-5MS capillary column (30 m, ID 250 μm, film thickness 0.25 μm; Part-No. 122-5532; Agilent). Lactic acid was quantified by monitoring its m/z ion ratio of 191. The calibration range for lactic acid was 0.3–33 μg per sample.

#### 2.7.4 Quantification of beta-alanine concentration by high-performance liquid chromatography

Extracellular beta-alanine concentration was analysed by ultra-performance liquid chromatography (UPLC). 250 μl of cell culture supernatant was deproteinized by adding 750 μl of ethanol (99.5%), the samples were mixed and centrifuged. The supernatant was transferred to a new vial and the samples were concentrated under a stream of N_2_. Finally, the volume was adjusted to 80 and 20 μl of borate buffer (MassTrak™, Waters) was added. 10 μl of the sample solution was analysed. The internal standard solution (norvaline, 25 μM), MassTrak™ Amino Acid Analysis (AAA) borate buffer and 6-aminoquinolyl-N-hydroxysuccinimidyl carbamate reagent were added, and sample mixture was instantly vortexed before incubation at 55°C for 10 min. Amino acid standard mixtures were derivatized identically to samples.

UPLC analysis was performed using an Acquity UPLC system equipped with an UV detector (Waters, Milford, MA, United States). Chromatography was performed using an Acquity MassTrak™ (2.1 mm × 100 mm, 1.7 μm) column (Waters) and kept at 43°C. The injection volume was 1 μl. Separation was performed using gradient elution with 10% (v/v) MassTrak™ AAA eluent A concentrate in water and MassTrak™ AAA eluent B at a flow rate of 0.4 ml min^−1^ using a gradient elution program. The signal for beta-alanine was detected at 260 nm. MassTrak™ AAA derivatization kit, Mass TRAK™ AAA concentrate A and eluent B were obtained from Waters. Amino acid standard solution, physiological amino acid standards, L-isoleucine, glutamine, and norvaline were obtained from Sigma-Aldrich (St. Louis, MO).

## 3 Results

### 3.1 Genome analysis of *R. opacus* DSM 43205

**FIGURE 2:**
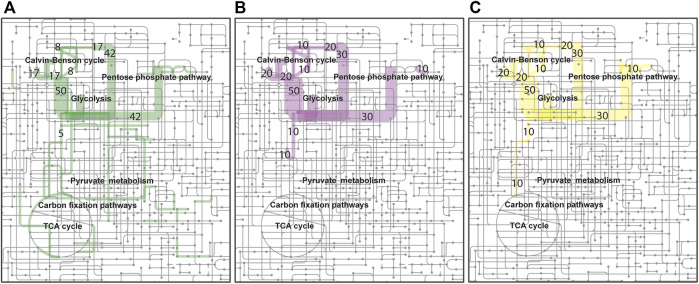
Autotrophic, H_2_ oxidizing, metabolism of *R. opacus* DSM 43205. Flux distributions of optimal H_2_ limited **(A)** growth (green), **(B)** lactic acid (purple), and **(C)** beta-alanine (yellow) production simulated using the reconstructed genome-scale metabolic model for *R. opacus* DSM 43205 are shown overlaid on the KEGG map of Microbial metabolism (id: map01120). The colored line widths indicate the flux magnitudes as relative fluxes normalized to arbitrary limiting H_2_ utilization of 100 mmol/(g CDW h) [≤4 mmol/(g CDW h) with same narrow width] with flux numbers for the highest fluxes.

The genome sequence of *R. opacus* DSM 43205 was determined by combined short- and long-read sequencing in order to obtain a data of sufficient sequencing coverage and accuracy that allows identification of the autotrophy-related genes and a construction of a metabolic model. The Illumina data set contained over 5.53 million paired-end reads, with lengths that ranged from 50 to 126. The PacBio-based long read data set contained over 758 × 10^3^ reads with lengths that ranged from 50 to 41,685. The Unicycler-based genome assembly contained eighteen contigs (length >1,500 bases) together representing a genome of 89,42,682 bases. The largest contig (and N50) was 6,484,583 bases long. The GC content of the assembly was 67%. A number of (*n* = 8,418) gene coding sequences (CDS) were found in the genome assembly. Of these, majority of the CDS (*n* = 5,999) were found on the largest contig. A number of the sequences (*n* = 2,684) were identified as metabolic enzymes with known EC numbers, which mapped to over 145 metabolic pathways in the Kyoto Encyclopedia of Genes and Genomes (KEGG) database. The pathway coverage of metabolic enzymes was found similar to *R. opacus* PD630 (Supplementary Material S1; Figure 1). The gene encoding RuBisCo (EC:4.1.1.39) was found in the genome assembly (on the fifth largest contig) confirming the presence of the CBB cycle, along with the hydrogenase genes (NAD-reducing hydrogenase HoxS subunits, EC: 1.12.1.2 in contigs 2, and 5) (Supplementary Material S1; Figure 2). Together, these markers confirmed the experimentally observed CO_2_ metabolism of the *R. opacus* DSM 43205 strain and suggest that the fifth contig of the genome assembly represents the autotrophic plasmid pHG201 described earlier (Kalkus et al., 1990; Grzeszik et al., 1997; Schwartz, 2009).

### 3.2 Genome-scale metabolic model simulations

Automatically reconstructed genome-scale metabolic model of *R. opacus* DSM 43205 (containing 2,267 reactions and 1,499 metabolites) was manually curated for aerobic autotrophic growth with soluble hydrogen hydrogenase with oxygen as the final electron acceptor. The model-predicted that the H_2_–CO_2_ utilization ratio for optimal growth was 4.11 mol H_2_ per mol CO_2_ without considering H_2_ oxidization required for growth rate -independent maintenance. At higher ratio, the growth was predicted to be limited by the availability of CO_2_, whereas at lower utilization ratio the growth was predicted to be H_2_-limited. Model simulations were performed also for predicting H_2_-CO_2_ utilization ratios required for optimal lactic acid and beta-alanine production by *R. opacus* DSM 43205. The H_2_ to CO_2_ utilization ratios for the optimal synthesis of these compounds were predicted with model simulations very similar for the two products and slightly lower than for growth. The predicted utilization ratios were 3.07 mol H_2_ per mol CO_2_ and 3.19 mol H_2_ per mol CO_2_ for L-lactic acid and beta-alanine, respectively. The predictions did not consider growth rate-independent requirement of H_2_ oxidation for maintenance energy. The required maintenance energy would be expected to cause equal additive increase in the H_2_ utilization for growth and production but would depend on the extracellular conditions (Bongers, 1970). Thus, if the cells utilized H_2_ and CO_2_ as optimal for growth, CO_2_ fixation would limit the production. Based on the simulations, there were no large differences in the H_2_–CO_2_ utilization ratio for optimal growth, nor for production of either of the products. A schematic figure (Figure 2) shows also how the flux distributions in optimal growth, beta-alanine, and lactic acid production are notably similar excluding the biosynthetic fluxes that are low relative to the central metabolic fluxes. Therefore, the cultivations were performed in identical gas feeding ratios. Thus, in addition to suggesting beneficial genetic modifications, this manually curated model can help in designing the experimental set-up.

### 3.3 Heterotrophic and autotrophic L-lactic acid production

**FIGURE 3:**
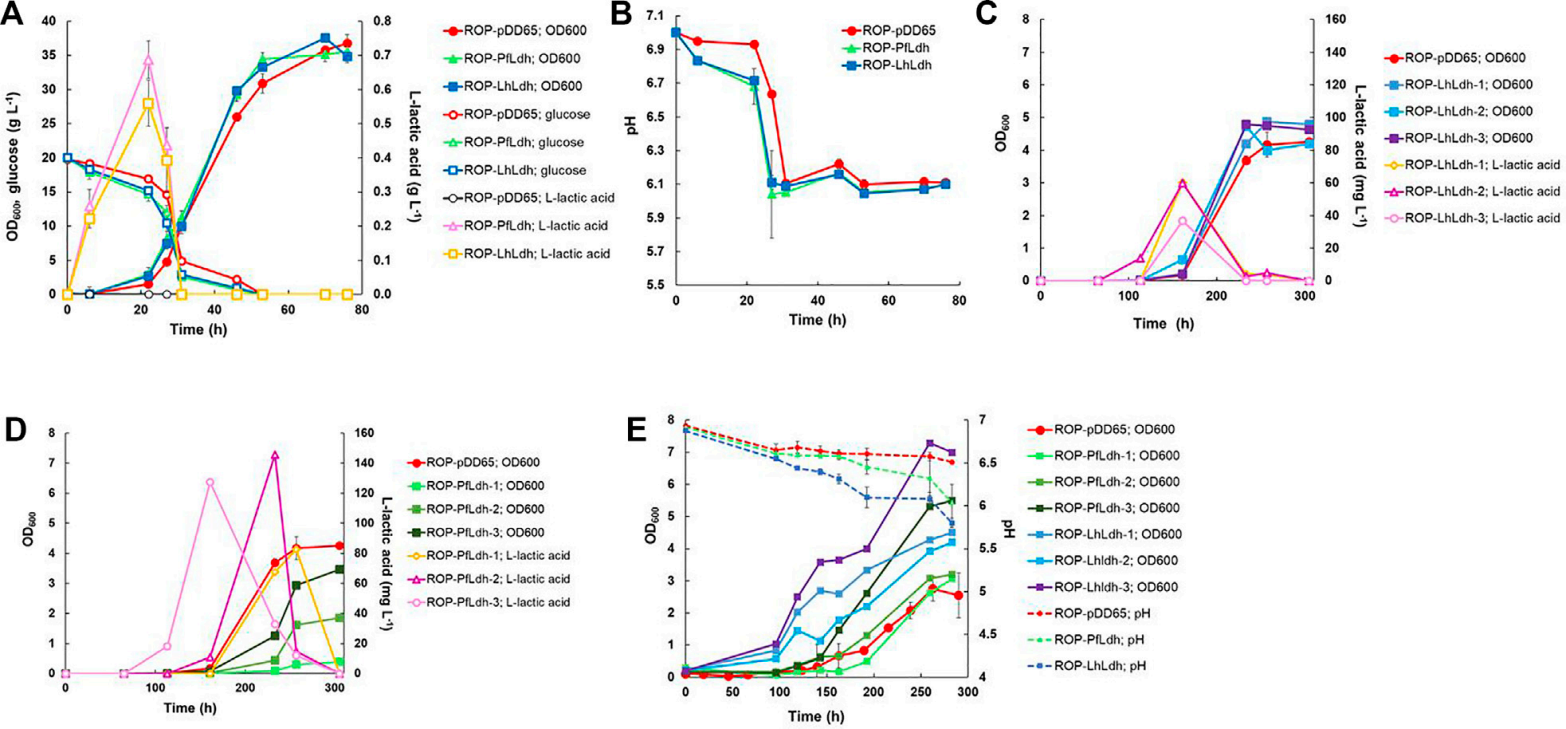
Heterotrophic and autotrophic production of L-lactic acid and biomass by derivatives of *R. opacus* strain DSM 43205 containing genes encoding either a lactate dehydrogenase of *L. helveticus* (Lh*ldh*) or *P. falciparum* (Pf*ldh*). Heterotrophic shake-flask cultivations **(A,B)** were performed in DSM-81 medium supplemented with 20 g L^−1^ glucose and glucose and lactic acid concentration as well as cell density (OD_600nm_) **(A)** and pH were monitored **(B)**. Subfigures **(A)** and **(B)** display average of biological triplicates with standard deviations. L-lactic acid concentration, pH change, and cell growth were monitored during autotrophic cultivations of transformants of *R. opacus* strains ROP-LhLdh **(C)** and ROP-PfLdh **(D)** and, due to large differences between the individual measurements, are displayed in separate subfigures and as individual measurements for clarity. Changes in cell density (OD_600nm_) and pH during autotrophic electrobioreactor cultivations of *R. opacus* strains ROP-LhLdh and ROP-PfLdh are shown in subfigure **(E)** *R. opacus* pDD65 is included as a control strain and data points represent averages of triplicate measurements with error bars indicating the standard deviation.

L-lactate production was studied by expressing genes encoding L-lactate dehydrogenases of *P. falciparum* (Pf*ldh*) and *L. helveticus* (Lh*ldh*) in *R. opacus* DSM 43205. These enzymes have different kinetic properties and they both have been expressed before for heterologous L-lactic production (Novy et al., 2017). L-lactate dehydrogenases were cloned into the expression plasmid pDD57 where their expression was controlled by a constitutive promoter of *S. lividans* TK24. *R. opacus* DSM 43205 was transformed with either the pDD57 construct harbouring Pf*ldh* or Lh*ldh* yielding the strains ROP-PfLdh and ROP-LhLdh, respectively. First, in order to confirm the activity of expressed L-lactate dehydrogenases in *R. opacus*, L-lactic acid production by three ROP-PfLdh and three ROP-LhLdh transformants was studied in shake flask cultures supplemented with 20 g L^−1^ glucose. The *R. opacus* strains ROP-PfLdh and ROP-LhLdh produced up to 742 mg L^−1^ and 608 mg L^−1^ L-lactic acid at rates of 43 ± 8 mg L^−1^ h^−1^ and 37 ± 4 mg L^−1^ h^−1^ and specific productivities of 20 ± 2 mg g CDW^−1^ h^−1^ and 17 ± 2 mg g CDW^−1^ h^−1^, respectively (Figure 3A; Table 2). During both cultivations, L-lactic acid accumulated at the beginning of the cultivations during the 20 h phase when both, glucose consumption and biomass accumulation occurred at low rates. During the following 10 h, cells rapidly consumed the produced L-lactic acid and almost all glucose and accumulated biomass until 53 h as indicated by an increase of OD_600nm_ up to 35. The pH in the cultivations of the L-lactic acid producing strains were lower compared with the control strain with the empty expression vector during the L-lactic acid production phase but did not remarkably differ from the control at the later stages of cultivations (Figure 3B). Interestingly, the L-lactic acid producing strains consumed glucose slightly faster and reached a somewhat higher biomass than the control strain. The control strain ROP-pDD65 with the empty plasmid did not produce L-lactic acid. Homology search of the genome sequence of *R. opacus* DSM 43205 did not suggest the presence of L-lactate dehydrogenase in *R. opacus* that is in accordance with its inability to produce L-lactic acid. Instead, *R. opacus* DSM 43205 genome has homologs for L-lactate cytochrome c reductase that links lactate oxidation to electron transfer to mitochondrial respiratory chain (Ingledew and Poole, 1984), for *lut*ABC operon that is present in genomes of various bacteria and is linked to lactate utilization and biofilm formation (Chai et al., 2009) and for lactate-2-mono-oxygenase (Kean and Karplus, 2019).

**TABLE 2:** Volumetric and specific L-lactic acid and beta-alanine production rates of *R. opacus* strains measured during autotrophic electrobioreactor (EB-CO_2_) and shake flask cultivations with either glucose (SF-Glc) or CO_2_ (SF-CO_2_) as carbon source. In all performed EB-CO_2_ trials, L-lactic acid concentrations remained below the detection limit and are not shown here. The shown data is determined from triplicate measurements (i.e., three individual transformant cultivations per strain) and the CDW-specific productivity was determined from data recorded from the cultivation start until the time of measured maximum product concentration (see Figures 3, 4).

*R. opacus* strain	Cultivation mode	Productivity [mg L^−1^ h^−1^]	Glc-specific yield [mg g^−1^]	CDW-specific productivity [mg g^−1^ h^−1^]	CDW-specific yield [mg g^−1^ ]
		L-lactic acid production			
ROP-PfLdh	SF-Glc	43.1 ± 8.1	138.4 ± 11.1	19.8 ± 1.7	436.9 ± 37.2
ROP-PfLdh	SF-CO_2_	0.58 ± 0.24	—	0.64 ± 0.28	129.2 ± 34.5
ROP-LhLdh	SF-Glc	36.9 ± 4.4	112.7 ± 13.7	16.8 ± 1.9	369.6 ± 43.1
ROP-LhLdh	SF-CO_2_	0.33 ± 0.08	—	0.34 ± 0.08	55.5 ± 13.2
		Beta-alanine production			
ROP-PanD	SF-Glc	0.66 ± 0.012	1.63 ± 0.27	0.22 ± 0.002	6.73 ± 0.05
ROP-PanD	SF-CO_2_	0.01 ± 0.002	—	0.01 ± 0.003	1.93 ± 0.42
ROP-PanD	EB-CO_2_	0.0016 ± 0.0001	—	0.00076 ± 0.00002	0.16 ± 0.004

Next, L-lactic acid production from CO_2_ was investigated. Both strains, ROP-PfLdh and ROP-LhLdh were grown under autotrophic conditions in shake flask cultures under a gas atmosphere with the composition of 49% N_2_, 25% CO_2_, 13% H_2_, and 13% O_2_. Growth of the control strain and ROP-PfLdh and ROP-LhLdh initiated after ∼100 h after which L-lactic acid accumulated in the culture supernatant to concentrations of up to 146 mg L^−1^ and 61 mg L^−1^, respectively (Figures 3C,D). Especially in case of ROP-PfLdh, there was a large variation in growth and L-lactic acid production between the transformants studied (Figure 3D). The specific L-lactic acid productivities of both *R. opacus* strains ROP-PfLdh and ROP-LhLdh under autotrophic growth conditions were 0.6 ± 0.3 mg g CDW^−1^ h^−1^ and 0.3 ± 0.1 mg g CDW^−1^ h^−1^, respectively, and significantly lower than those that were measured when glucose was used as a carbon source (Table 2). Similar to heterotrophic cultivations, the highest L-lactic acid accumulation occurred during the early exponential growth phase of strains ROP-PfLdh and ROP-LhLdh. Notably, ROP-LhLdh produced less L-lactic acid and more biomass than ROP-PfLdh while transformants of the latter strain accumulated less biomass and produced over two fold more L-lactic acid. In contrast, no significant difference was observed in growth and L-lactic acid production between the strains ROP-PfLdh and ROP-LhLdh during the heterotrophic cultivations (Figure 3A).

Finally, characteristics of the strains and L-lactic acid production was studied in electrobioreactors with 20% CO_2_ introduced into the cultivation broth by sparging. H_2_ and O_2_ were synthesized *in situ* by water electrolysis that was performed at a constant current of 18 mA. Under these conditions, both ROP-PfLdh and ROP-LhLdh exhibited cell growth (Figure 3E) but L-lactic acid accumulation could not be detected. The ROP-LhLdh transformants grew to higher cell densities than the control strain similar to shake flask cultures that were grown with CO_2_ as carbon source. pH of the fermentations was followed regularly and the drop of pH correlated with growth of the strains. The final pH of all fermentations remained above pH 5.5 (Figure 3E).

### 3.4 Heterotrophic and autotrophic beta-alanine production

**FIGURE 4:**
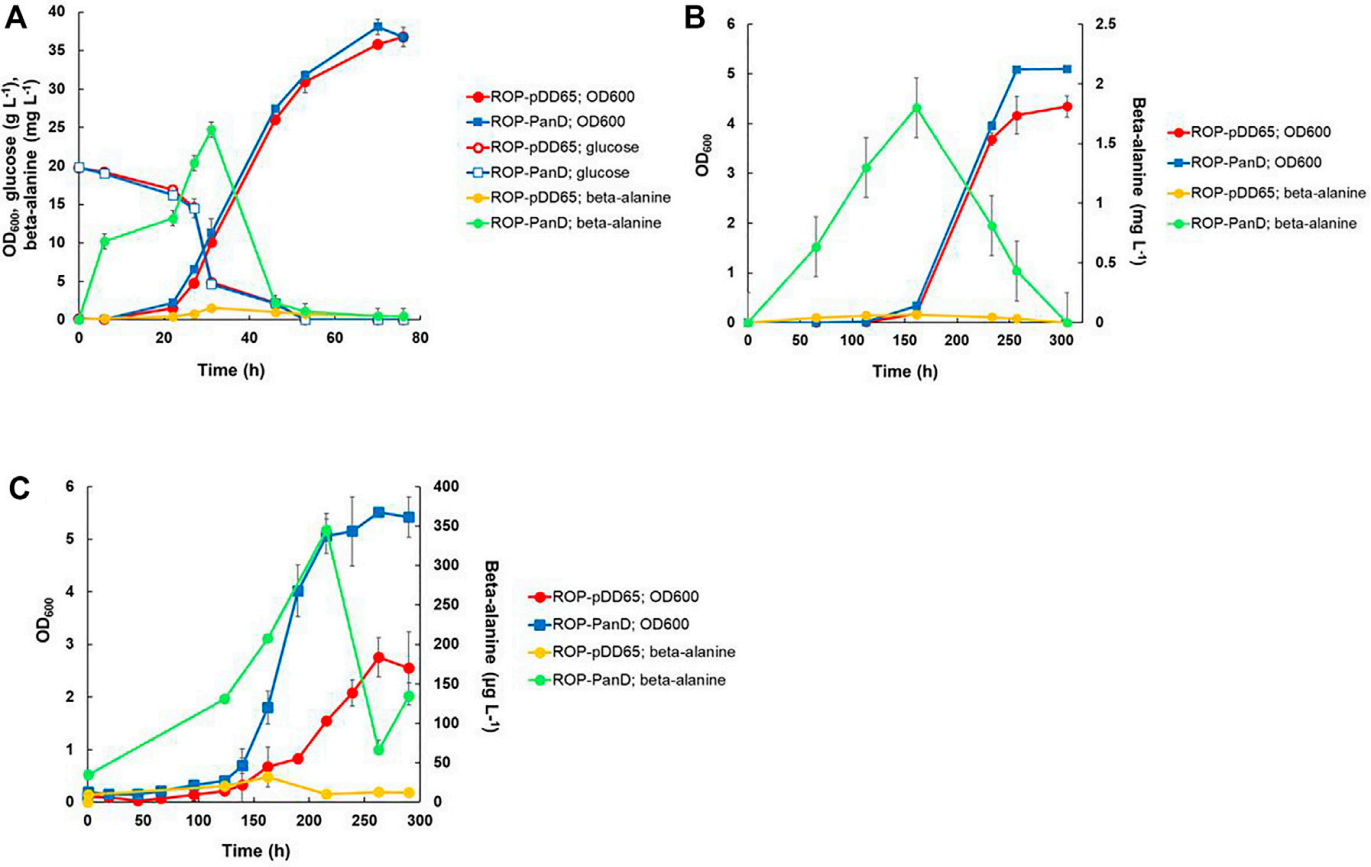
Heterotrophic and autotrophic shake-flask cultivation [**(A,B)**, respectively] and autotrophic electrobioreactor cultivation **(C)** of transformants of *R. opacus* strain ROP-PanD expressing the aspartate decarboxylase gene of *C. glutamicum* (*panD*). Beta-alanine concentration and cell density (OD_600_) were followed during the course of the cultivations and displayed as average of biological triplicates with standard deviation. *R. opacus* pDD65 is included as a control strain (ctrl).

The gene encoding aspartate 1-decarboxylase (*panD*) of *C. glutamicum* that showed relatively good beta-alanine productivity in heterotrophic production in *E. coli* (Song et al., 2015) was cloned into the expression plasmid pDD57 and placed under the control of a constitutive promoter of *S. lividans* TK24. *R. opacus* DSM 43205 was transformed with this vector resulting in *R. opacus* strain ROP-panD. *R. opacus* strain ROP-panD and the control strain ROP-pDD65 exhibited very similar growth profile during heterotrophic cultivation on glucose (Figure 4A) and reached an OD_600nm_ of up to 38. The beta-alanine concentration in the culture broth of *R. opacus* strain ROP-PanD reached 25 mg L^−1^ corresponding to a specific productivity of 0.2 ± 0.002 mg g CDW^−1^ h^−1^. Notably, in glucose cultivations beta-alanine production was also detected in the control experiment with strain pDD65 yielding product concentrations of up to 1.6 mg L^−1^. In both cases, beta-alanine concentration increased during the early growth phase when glucose was still consumed at a relatively low rate. Thereafter, during late exponential growth phase, the beta-alanine concentration rapidly decreased by the end of the cultivation.

Beta-alanine concentration of shake flask cultivations of *R. opacus* ROP-PanD reached maximum values of 1.8 mg L^−1^ when the energy and carbon substrates were provided in gaseous form (49% N_2_, 25% CO_2_, 13% H_2_, and 13% O_2_) (Figure 4B). The specific beta-alanine productivity of autotrophic shake flask cultivations was 0.01 ± 0.003 mg g CDW^−1^ h^−1^, which corresponded to just ∼5% of the value measured for the heterotrophically grown strain (Table 2). It is noteworthy that, during the provision of CO_2_ as an exclusive carbon source, beta-alanine was produced during a ca. 100 h lag phase before growth initiation and it was then consumed during the subsequent growth phase as apparent by observed reduction of product concentration. Beta-alanine producing transformants reached 17% higher OD_600nm_ values than the control strains (OD_600nm_ 5 vs. 4.2). In contrast to L-lactic acid, beta-alanine production was demonstrated also in electrobioreactor sparged with 20% CO_2_ and with provision of H_2_ and O_2_ by water electrolysis at a constant current of 18 mA. Under these conditions, ROP-PanD strains produced 345 μg L^−1^ beta-alanine from CO_2_ (Figure 4C). Beta-alanine started to accumulate already during the lag phase of the cultivations, but the highest production coincided with the onset of the exponential growth phase. Subsequently, the produced beta-alanine was consumed from the media. ROP-panD strains reached 50% higher biomass (OD_600nm_ = 5.5) than the control strains.

## 4 Discussion

*R. opacus* strain DSM 43205 is an interesting representative of lesser-known aerobic facultative chemolithotrophs that could potentially be used for CO_2_-based chemical synthesis (Aggag and Schlegel, 1974; Brigham, 2019). In the present study, we sequenced the genome of *R. opacus* strain DSM 43205, build a model of its metabolism, and genetically engineered it for the conversion of either CO_2_ or glucose to the biopolymer precursor beta-alanine or L-lactic acid. The latter was done by expression of recombinant genes encoding either aspartate decarboxylase or L-lactate dehydrogenase, respectively. Beta-alanine production from CO_2_ was demonstrated also in electrobioreators where H_2_ and O_2_ were provided by *in situ* water electrolysis. Use of H_2_ as a gaseous substrate in fermentations can be difficult due to its low solubility and flammability in the presence of oxygen at an oxidant concentration of 4%–94%. The *in situ* water electrolysis for hydrogen provision that was employed here could be considered as a sustainable and safe alternative to the conventional provision of H_2_ gas from large reservoirs located outside of the bioreactor.

Beta-alanine is a precursor of pantothenate and coenzyme A biosynthesis and its biosynthesis involves at least three enzymatic reactions from phosphoenolpyruvate depending on whether aspartate is formed directly from oxaloacetate or through the TCA cycle (Piao et al., 2019) (Figure 1). The sequenced *R. opacus* genome encodes genes for both of these routes. It also possesses an endogenous gene for aspartate decarboxylase and a small amount of beta-alanine was produced in both glucose and CO_2_ cultivations with the control strain. Overexpression of *panD* from *C. glutamicum* increased beta-alanine production significantly from both carbon sources. Interestingly, ROP-PanD strains produced also more biomass than the control from both glucose and CO_2_, especially in electrobioreactor cultivations. There is no evident reason for this but possibly consumption of the produced beta-alanine from the growth medium boosted the carbon metabolism and growth. Moreover, decarboxylation of aspartate to beta-alanine releases intracellular CO_2_ that may be more readily available as a substrate for the CBB cycle than the extracellular CO_2_ that is sparged into the growth medium.

No L-lactic acid production could be observed during hetero- or autotrophic cultivation of *R. opacus* strain DSM 43205. The production of L-lactic acid requires a single enzymatic step catalysing the reduction of the central carbon metabolite pyruvate. Here, we overexpressed L-lactate dehydrogenase genes from *L. helveticus* (Lh*ldh*) and *P. falciparum* (Pf*ldh*) with different catalytic properties in *R. opacus*. PfLdh has a significantly higher catalytic efficiency and affinity for pyruvate (*K*_*m*_ = 0.03 mM) than LhLdh (*K*_*m*_ = 0.25 mM) (Novy et al., 2017). ROP-PfLdh strains produced almost 2.5 times the amount of L-lactic acid from CO_2_ in shake flask cultivations compared to ROP-LhLdh strains, which may be attributed to the high substrate affinity of PfLdh. PfLdh had also higher specific L-lactic acid productivity due to lower biomass production. Interestingly, both strains produced almost equal amounts of L-lactic acid from glucose, which is possibly due to a higher intracellular pyruvate concentration under these conditions.

Surprisingly, during electrobioreactor cultivations no L-lactic acid production could be detected, although biomass production did not differ much from the autotrophic shake flask cultivations. Likewise to autotrophic shake flask cultivations, ROP-LhLdh strains grew to higher cell densities than control and ROP-PfLdh strains. It is possible that in small electrobioreactors water splitting with relatively low current resulted in limited generation of H_2_ and produced L-lactic acid was possibly even more readily utilized by the cells for carbon and redox supply than in autotrophic shake flask cultures.

Beta-alanine and L-lactic acid offered a possibility to study chemical production from CO_2_ from different metabolic routes; L-lactic acid being linked directly to central carbon metabolism and beta-alanine being a product from amino acid metabolism. Despite the difference in length of L-lactic acid and beta-alanine biosynthetic routes from pyruvate, the production of both compounds was observed already during the lag and early exponential growth phase, independent on whether glucose or CO_2_ was available as the carbon source. Especially, the L-lactic acid production appeared to increase the initial glucose consumption rate in the beginning of the cultivations and was followed by a consumption of both L-lactic acid and beta-alanine. There is no clear explanation for a long lag phase of growth observed on both carbon sources and especially when cells were grown on CO_2_. Kim et al., 2019 examined the long 48 h lag phase of *R. opacus* PD630 in glucose fermentation and demonstrated that decrease of cultivation pH from 7.0 to 6.4 reduced the lag phase significantly to 24 h (Kim et al., 2019).

However, economically viable production titers would require more elaborate metabolic engineering efforts. As an example, in *E. coli,* efficient production of beta-alanine from glucose requires overexpression of all genes encoding the enzymes of the reductive branch of TCA cycle and deletion of several pathways for side products (Piao et al., 2019; Zou et al., 2020). In *E. coli*, the uptake of beta-alanine is performed by an active amino acid transporter (Schneider et al., 2004). Deletion of the corresponding transporter present in *R. opacus* genome could possibly prevent the beta-alanine utilization from the surrounding growth medium. Likewise, improved L-lactic acid production would require more extensive metabolic engineering as exemplified by work carried out with *E. coli* (Jiang et al., 2017). The sequencing of the genome of *R. opacus* DSM 43205 revealed the presence of open reading frames with the homology to L-lactate cytochrome c reductase, to *lut*ABC operon and to lactate-2-mono-oxygenase, all involved in lactate catabolism observed in our cultivations (Ingledew and Poole, 1984; Chai et al., 2009; Kean and Karplus, 2019). However, the inefficient homologous recombination in *R. opacus* hampered our efforts to delete these open readings from the genome despite the use of sacB counter selection and the help of bacteriophage recombinases described and used in earlier studies (Kita et al., 2009; DeLorenzo et al., 2018). Recently, fast-growing interest in metabolic versatility and use of *R. opacus* as a platform for biocatalysis, biodegradation and biosynthesis has driven development of more genome engineering tools for *R. opacus*. New genetic parts have been validated and most importantly CRISPR/Cas9-based gene knockout system is reported (Liang et al., 2021; Grechishnikova et al., 2022). These tools are anticipated to facilitate and accelerate the engineering of *R. opacus* DSM 43025 to yield outstanding strains for the production of biochemicals and biofuels from CO_2_.

Likewise, *C. necator*, the most well-known lithoautotroph, has been engineered for autotrophic production of various chemical compounds. The cultivation systems using shake flasks or classical bioreactor flushed with commercial gas mixtures with variable CO_2_, H_2_, and O_2_ concentrations demonstrated typically mg/L proof of concept production levels, comparable to L-lactic acid produced in present study (Müller et al., 2013; Grousseau et al., 2014; Przybylski et al., 2015; Crépin et al., 2016; Chakravarty and Brigham, 2018; Krieg et al., 2018; Windhorst and Gescher, 2019). The different cultivation conditions applied and in most cases compromised and growth limiting gas supply make the comparison of performance of *R. opacus* DSM 43205 to *C. necator* production strains difficult. The recent studies with *C. necator* indicated the importance of optimizing the gas fermentation process and bioreactor design for improved product yield (Garrigues et al., 2020). This enables also achievement of higher biomass and more realistic conditions for determination of product titers, productivity and yields.

The observed predominant assimilation of externally supplied CO_2_ into biomass instead of products, motivates further metabolic studies of *R. opacus* DSM 43025 that may enhance its industrial applicability for chemical production. The genome sequence refinement and genome-scale model reconstruction promote the discovery of further engineering strategies, including *in silico* strain design (Fang et al., 2020) for *R. opacus* DSM 43025 and provide data needed for its development into a potent autotrophic production host.

## 5 Conclusion

In summary, we have shown that the hydrogen-oxidizing bacterium *R. opacus* DSM 43205 can be engineered to synthesize value-added compounds using CO_2_ as exclusive carbon and hydrogen as energy source. Both, conventional gas fermentation and electrobioreactor cultivation using *in situ* water electrolysis where found suitable for carbon assimilation to desired products with engineered strains of *R. opacus* DSM 43205, but attenuation of product formation was observed in the electrobioreactor cultivations. The novel hosts resulting from the work contribute to the transition from CO_2_-releasing manufacture of chemicals to CO_2_-fixing bioprocesses. The cultivation methods and metabolic models developed in this project will facilitate further studies of still mostly unexplored lithoautotrophic microbial species.

## Data Availability

The datasets presented in this study can be found in online repositories. The names of the repository/repositories and accession number(s) can be found in the article/Supplementary Material.

## Abbreviations

CBB, Calvin-Benson-Bassham; ATP, adenosine triphosphate.
