# Supplementary material for: Production of biopolymer precursors beta-alanine and L-lactic acid from CO2 with metabolically versatile Rhodococcus opacus DSM 43205
Source: Front Bioeng Biotechnol. 2022 Oct 7;10:989481. doi: 10.3389/fbioe.2022.989481 (PMC9587121; doi:10.3389/fbioe.2022.989481)
Supplement: Supplementary file 1 [file DataSheet1.docx]

Additional file 1

# Production of biopolymer precursors beta-alanine and lactic acid from CO_2_ with metabolically versatile *Rhodococcus opacus*

Table 1. List of optimized gene sequences.

| Gene | Nucleotide sequence |
| --- | --- |
| *panD* | ATGCTCCGCACCATCCTCGGCTCCAAGATCCACCGCGCCACCGTCACCCAGGCCGACCTCGACTACGTCGGCTCCGTCACCATCGACGCCGACCTCGTCCACGCCGCCGGCCTCATCGAGGGCGAGAAGGTCGCCATCGTCGACATCACCAACGGCGCCCGCCTCGAGACTTACGTCATCGTCGGCGACGCCGGCACCGGCAACATCTGCATCAACGGCGCCGCCGCCCACCTCATCAACCCCGGCGACCTCGTCATCATCATGTCCTACCTCCAGGCCACCGACGCCGAGGCCAAGGCCTACGAGCCCAAGATCGTCCACGTCGACGCCGACAACCGCATCGTCGCCCTCGGCAACGACCTCGCCGAGGCCCTCCCCGGCTCAGGCCTCCTCACCTCCCGCTCCATCTAA |
| Pf*ldh* | ATGGCCCCCAAGGCCAAGATCGTCCTCGTCGGCTCCGGCATGATCGGCGGCGTCATGGCCACCCTCATCGTCCAGAAGAACCTCGGCGACGTCGTCCTCTTCGACATCGTCAAGAACATGCCCCACGGCAAGGCCCTCGACACCTCCCACACCAACGTCATGGCCTACTCCAACTGCAAGGTATCCGGCTCCAACACCTACGACGACCTCGCCGGCGCCGACGTCGTCATCGTCACCGCCGGCTTCACCAAGGCCCCCGGCAAGTCCGACAAGGAGTGGAACCGCGACGACCTCCTCCCCCTCAACAACAAGATCATGATCGAGATCGGCGGCCACATCAAGAAGAACTGCCCCAACGCCTTCATCATCGTCGTCACCAACCCCGTCGACGTCATGGTCCAGCTCCTCCACCAGCACTCCGGCGTCCCCAAGAACAAGATCATCGGCCTCGGCGGCGTCCTCGACACCTCCCGCCTCAAGTACTACATCTCCCAGAAGCTCAACGTCTGCCCCCGCGACGTCAACGCCCACATCGTCGGCGCCCACGGCAACAAGATGGTCCTCCTCAAGCGCTACATCACCGTCGGCGGCATCCCCCTCCAGGAGTTCATCAACAACAAGCTCATCTCCGACGCCGAGCTCGAGGCCATCTTCGACCGCACCGTCAACACCGCCCTCGAGATCGTCAACCTCCACGCCTCCCCCTACGTCGCCCCCGCCGCCGCCATCATCGAGATGGCCGAGTCCTACCTCAAGGACCTCAAGAAGGTCCTCATCTGCTCCACCCTCCTCGAGGGCCAGTACGGCCACTCCGACATCTTCGGCGGCACCCCCGTCGTCCTCGGCGCCAACGGCGTCGAGCAGGTCATCGAGCTCCAGCTCAACTCCGAGGAGAAGGCCAAGTTCGACGAGGCCATCGCCGAGACTAAGCGCATGAAGGCCCTCGCCTAA |
| Lh*ldh* | ATGGCCCGCGAGGAGAAGCCCCGCAAGGTCATCCTCGTCGGCGACGGCGCCGTCGGCTCCACCTTCGCCTTCTCCATGGTCCAGCAGGGCATCGCCGAGGAGCTCGGCATCATCGACATCGCCAAGGAGCACGTCGAGGGCGACGCCATCGACCTCGCCGACGCCACCCCCTGGACCTCCCCCAAGAACATCTACGCCGCCGACTACCCCGACTGCAAGGACGCCGACCTCGTCGTCATCACCGCCGGCGCCCCCCAGAAGCCCGGCGAGACTCGCCTCGACCTCGTCAACAAGAACCTCAAGATCCTCTCCTCCATCGTCGAGCCCGTCGTCGAGTCCGGCTTCGAGGGCATCTTCCTCGTCGTCGCCAACCCCGTCGACATCCTCACCCACGCCACCTGGCGCATGTCCGGCTTCCCCAAGGACCGCGTCATCGGCTCCGGCACCTCCCTCGACACCGGCCGCCTCCAGAAGGTCATCGGCAAGATGGAGAACGTCGACCCCTCCTCCGTCAACGCCTACATGCTCGGCGAGCACGGCGACACCGAGTTCCCCGCCTGGTCCTACAACAACGTCGCCGGCGTCAAGGTCGCCGACTGGGTCAAGGCCCACAACATGCCCGAGTCCAAGCTCGAGGACATCCACCAGGAGGTCAAGGACATGGCCTACGACATCATCAACAAGAAGGGCGCCACCTTCTACGGCATCGGCACCGCCTCCGCCATGATCGCCAAGGCCATCCTCAACGACGAGCACCGCGTCCTCCCCCTCTCCGTCCCCATGGACGGCGAGTACGGCCTCCACGACCTCCACATCGGCACCCCCGCCGTCGTCGGCCGCAAGGGCCTCGAGCAGGTCATCGAGATGCCCCTCTCCGACAAGGAGCAGGAGCTCATGACCGCCTCCGCCGACCAGCTCAAGAAGGTCATGGACAAGGCCTTCAAGGAGACTGGCGTCAAGGTCCGCCAGTAA |

Figure 1. Metabolic capabilities of *R. opacus* DSM 43205. A) Metabolic enzyme annotations of *R. opacus* DSM 43205 (orange) obtained from the genome assembly overlayed with R. opacus PD630 (blue) annotations on KEGG map of Microbial metabolism (id:map01120). The metabolic reactions covered with annotations in both genomes are shown in overlaid orange and blue colors (brown).

Figure 2. RuBiSCo and hydrogenases found in the *Rhodococcus opacus* DSM 43205 assembly confirming the hydrogen oxidizing autotrophic activity. The largest seven contigs are shown in the picture with markings of where RuBiSCo (green color labels) and hydrogenases (red color labels) were found. Note that the NAD-reducing hydrogenase HoxS subunits (EC: 1.12.1.2) found on contigs 2 and 5 are relevant for HOA activity, but not the periplasmic NiFeSe hydrogenaes subunits found on contig 1.
